# Influence of Measurement Uncertainty in the Determination of Selected Rock Parameters—A Realistic Approach

**DOI:** 10.3390/ma16083045

**Published:** 2023-04-12

**Authors:** Klaus Voit, Oliver Zeman, Peter Gappmaier, Karin Wriessnig, Renata Adamcova

**Affiliations:** 1 Institute of Applied Geology, University of Natural Resources and Life Sciences, Vienna, Peter Jordan-Straße 82, 1190 Vienna, Austria; 2 Institute of Structural Engineering, University of Natural Resources and Life Sciences, Vienna, Peter Jordan-Straße 82, 1190 Vienna, Austria; 3 Faculty of Natural Sciences, Comenius University in Bratislava, Ilkovicova 6, 842 15 Bratislava, Slovakia

**Keywords:** hard rock characterization, rock properties, field tests, rebound/Schmidt hammer, point load test, Los Angeles test, aleatoric and epistemic measurement uncertainty

## Abstract

To determine and compare the measurement uncertainty of different geological-geotechnical testing methods, numerous test locations were selected in a hard rock quarry. Measurements were carried out along two vertical measurement lines perpendicular to the mining levels of an existing exploration. Along these lines, the rock quality basically varies due to weathering (decreasing influence with increasing distance from the original ground surface), but also due to the influence of the geological-tectonic conditions on site. The mining conditions (blasting) are identical over the considered area. The rock quality was examined as follows: as field tests, the rock compressive strength was determined by means of point load test and rebound hammer, as laboratory method the Los Angeles test (standard laboratory test for the determination of the mechanical rock quality) was used to identify the impact abrasion resistance. The statistical evaluation and comparison of the results allowed conclusions to be drawn about the contribution of the individual test methods to the measurement uncertainty whereas, in practice, a priori information can be applied complementarily. It shows that the influence on the combined measurement uncertainty u of the different methods due to the geological variability in horizontal direction reaches values between 17 and 32%, whereby the rebound hammer method shows the highest values. However, the highest influences on the measurement uncertainties are a result of the vertical direction due to weathering phenomena with percentages of 55 to 70%. For the point load test, the vertical direction shows the highest significance with an influence of approximately 70%. This leads to the conclusion that a higher weathering degree of the rock mass shows an increasing effect on the measurement uncertainty which needs to be considered using a priori information in measurements.

## 1. General

### 1.1. Intent of Research

Rock classifications to assess the stability of rock in tunneling or slope construction are among the top disciplines in engineering geology [1,2,3]. In this context, rock strength plays a decisive role—in addition to the interface characteristics. The same applies to the estimation and monitoring of rock quality in quarries during the production of high-quality aggregates: methods for a basic characterization as well as for a continuous quality addressing are essential.

In principal, a basic distinction concerning different test methods for the determination of mechanical rock parameters—with different time and equipment requirements concerning the execution of the tests—can be made as follows [4]:

- field tests vs. laboratory tests;
- destructive vs. non-destructive methods;
- direct vs. indirect methods.

Field tests are portable and can be carried out quickly on site. As far as destructive methods are concerned, the rock or a rock fragment is required to test the mechanical strength and is destroyed in the course of the test (loading up to the breaking strength). By applying non-destructive testing methods, geophysical methods are used to determine the rock strength indirectly without destroying a sample. The distinction between direct and indirect methods again concerns the determination of a specific strength parameter via another rock parameter that can be determined more easily (e.g., the determination of the tensile strength and conversion to the rock compressive strength) [4,5].

There are numerous standard geological and geotechnical investigation methods that have proven themselves over several decades, such as the rebound (Schmidt) hammer (RH), point load test (PLT), and Los Angeles test (LAT) compared in the present research paper, e.g., [4,5,6].

There is also current research into the development of new methods for determining the mechanical properties and quality of rock mass [6,7,8]. In addition, further development is carried out via computer-based studies using distinct-element methods such as PFC when modeling without pre-defined discontinuities, e.g., [9,10,11] or UDEC [12,13,14], if the rock mass is mainly characterized by preestablished discontinuities and joints.

Simple, quick, and cheap on-site standard methods for the indirect characterization of the mechanical rock quality and estimation of the rock strength are still the rebound (Schmidt) hammer [15,16] and the point load test [17]. These sometimes differ considerably in terms of the methodological approach. The same applies to the conversions of the obtained basic values (e.g., the rebound value *R* or the corrected point load index *Is*_(50)_), into an unconfined compressive strength (UCS) based on empirical tests. Therefore, according to ISRM [18], it is necessary to speak of an estimated value (UCS*), if the uniaxial compressive strength determination (UCS) was not carried out on drill cores [19].

Nevertheless, the results of the different methods are often used synonymously to assess or describe the mechanical rock quality. The possible uncertainties regarding the reliability and comparability of the results are often underestimated, although these have already been published as implementation guidelines [18]. Therefore, this article deals with the determination of the “rock strength” via the common testing methods rebound hammer [15,16], point load test [17], and Los Angeles (*LA*) test [20] with regard to the determination of the measurement uncertainty for the individual testing methods. Due to the intense fracturing of the rock, an extraction of drill cores for the direct determination of the UCS was not possible.

The above-mentioned investigation methods, rebound hammer, point load test, and Los Angeles test, were applied simultaneously in numerous test locations in a hard rock quarry in Austria. The investigated rock is a granulite from the family of metamorphic rocks. Results are available in different numbers for the respective test. The measurements were carried out along two vertical measurement lines perpendicular to the mining levels. Along these lines, the rock quality basically varies due to weathering (decreasing influence with increasing distance from the original ground surface), but also due to the influence of the geological-tectonic conditions on site expressed as a horizontal variability. The mining conditions (blasting) are identical over the considered area. The objective is to assess the suitability for the intended use of the rock as aggregates and to consider the measurement uncertainties in the significance of the results.

### 1.2. Influences on Rock Material Parameters

From an engineering-geological point of view, a fundamental distinction is made between rock strength and rock mass strength. The rock strength is basically dependent on the type of rock (magmatic, metamorphic, sedimentary) and the minerals present, which depends on the weathering degree as well, e.g., [4]. The bigger the scale of observation, the more important are the discontinuities (e.g., joints) and weathering phenomena with regard to the rock mass strength [1,2]. For the evaluation of an aggregate (grain group or grain mixture) as in the present case, weathering as well as smaller scale foliation or stratification planes have a great influence on the rock strength. In contrast, dividing planes with a larger separation distance—if larger than the final grain diameter—play almost no significant role due to the processing of the rock material (blasting, crushing, sieving, washing). Basically, the following characteristics and influences affect the strength parameters of rock aggregates (compare [21]):

- rock type and mineralogy (for example, an igneous granite composed of quartz, feldspar and mica is usually stronger than limestone composed of calcite minerals);
- rock formation and genesis: tectonic stress during ductile deformation may lead to formation of a foliation, while a brittle deformation leads to the formation of joints and fissures [22];
- weathering: whereby the weathering starts preferentially at weak zones (e.g., interfaces), where agents such as water and air can penetrate the rock;
- type of material excavation and processing: possible damage due to microcracks (could not be observed for the examined rock by means of thin section analysis).

These parameters can vary spatially (horizontally and vertically) depending on the geological-tectonic conditions. As a result, the rock strengths can vary significantly within a very small scale. This circumstance can mainly be attributed to the presence of interfaces and a varying degree of weathering [23,24,25]. Weathering can affect both the interface itself and the surrounding rock. In terms of aggregate production, weathering of the interface surface is mostly unproblematic, but the progress of weathering into the surrounding rock results in a reduction in the rock (aggregate) strength. Starting from the surface level, weathering must always be considered as an influencing factor.

### 1.3. Contributions on Measurement Uncertainty

Measurement uncertainty can be described as the trustworthiness of numerical values/measurement results. This uncertainty or inaccuracy of the measured value is negatively influenced by errors. Uncertainty of measurement, therefore, is defined as “a parameter, which is associated with the result of a measurement, characterizing the dispersion of the values that could reasonably be attributed to the measurand” [26]. Therefore, the uncertainty of measurement expresses the estimation of the “error” of the measurement result differing from the “true value” of the measurement value.

According to JCGM 100:2008 [26], JCGM 104:2009 [27], and JCGM 200:2008 [28], two components are distinguished concerning the measurement error: (1) the ‘systematic error’ and (2) the ‘random error’. Systematic measurement errors can be quantified and are significant in terms of accuracy and a correction factor can be applied to identify this effect in the overall measurement scope. If performed this way, the systematic error reaches a value of zero. In contrast, a random error is the result of an unpredictable stochastic variation without the possibility of correction or compensation. This is only reduced by an increasing number of attempts [26].

When dealing with natural materials, the terms ‘aleatoric’ and ‘epistemic’ are used instead of ‘random error’ and ‘systematic error’. This is due to the fact, that the variability of the properties of natural complex materials, such as rock masses, defines by its origin an initial level of uncertainty. This is in contrast, for example, to building materials (e.g., concrete), whose production takes place under controlled conditions and which are basically homogeneous. This type of uncertainty cannot be removed due to it being a feature of the material itself and therefore leads to an ‘aleatoric’ uncertainty. ‘Epistemic’ uncertainty, on the other hand, refers to uncertainty caused by a lack of knowledge, for example the measures of a given material property. This type of uncertainty can be reduced by expanding the quantity and quality of data describing the considered property, e.g., [29].

#### 1.3.1. Uncertainty of Measurement

In [26], a concept of contributions to measurement uncertainty, covering both random/aleatoric and systematic/epistemic errors, is provided. However, the concept does not make a strict distinction between these two. Generally, the contribution is used in the way of a standard uncertainty format and is calculated as the result of the standard deviation or the coefficients of variation considering the number of samples.

#### 1.3.2. Standard Uncertainty

In [26], the contributing factors influencing the uncertainty of measurement are identified as type A and type B. These can be differentiated as follows: while type A is identified by statistical analysis of observation series—like the standard deviation (coefficient of variation) of the arithmetic mean value under the assumption of a normal distribution; type B is acquired by means of other methods than statistical analysis of observation series [26]. For type B, each measured value must be subject to a statistical distribution which allows the statistical parameters to be defined on the basis of the assumed distribution. Specifications for the distribution assumption can be preceding measurement data, experience or expertise in the behaviour. Information regarding the assumption of the distribution type may be: previous measurement data, experience or knowledge of the behaviour observed, specifications from manufacturers, literature or calibration procedure.

#### 1.3.3. Modelling the Measured Values

To characterize the input quantities on the measurement result according to [26], a modelling of measurement is re-quired, whereby the output quantity *Y* for the mathematical model is the measured value itself as stated in Equation (1):

(1) $$Y=f\left(X_{1},\ldots ,X_{N}\right)$$

If the true values of the quantities *X*_1_, …, *X_N_* of the measurement are unknown, the usage of estimated input values *x*_1_, …, *x_N_* is necessary (Equation (2)).

(2) $$y=f\left(x_{1},\ldots ,x_{N}\right)$$

According to [30], the modelling of the measurement should include the entire measurement chain, i.e., the entire measurement process, which obeys the principle of cause and effect.

#### 1.3.4. Combined Standard Uncertainty

Entry values ca may correlate or may not correlate to each other. Uncorrelated quantities are independent and have no relation with each other. The combined standard uncertainty is derived from the different single standard uncertainties (Equation (3)).

(3) $$u_{c}^{2}\left(y\right)=\sum_{i=1}^{n}{\left(\frac{\partial f}{\partial x_{i}}\right)}^{2}u^{2}\left(x_{i}\right)$$

Thereby, a sensitivity coefficient *c* accounting the influence of a single quantity on the combined measurement uncertainty is included and calculated according to Equation (4).

(4) $$c=\frac{\partial f}{\partial x_{i}}\underset{}{\Rightarrow }\frac{\Delta f}{\Delta x_{i}}$$

#### 1.3.5. Expanded Uncertainty of Measurement

The expanded uncertainty of measurement *U* is derived by the multiplication of the combined standard uncertainty *u_c_*(*y*) by a coverage factor *k* (Equation (5)), the latter defining the level of confidence for the measurement uncertainty based on a probabilistic approach. In [26] a coverage factor 2 ≤ *k* ≤ 3 is recommended, whereby *k* = 2 defines a level of confidence of approximately 95% and *k* = 3 of approximately 99%.

(5) $$U=k\cdot u_{c}\left(y\right)$$

## 2. Materials and Methods

### 2.1. Tested Rock Structure

The investigated rock site lies in the Bohemian Massif in Austria. The rock at present is a granulite from the Moldanubicum with an age of approximately 340 Ma, which underwent metamorphism during the Variscan Orogenesis at about 800 °C and a pressure of about 1800 MPa. The granulite was subjected to mylonitic foliation in the course of exhumation, as well as to scarping of the “bench-like” metamorphic structures [31,32]. The structure is mylonitic and is characterized by recrystallization processes, whereby an intensive interlocking of the individual minerals develops, which finally also contributes to the high strength of this rock. In the course of cooling (retrograde branch of metamorphism), a very subordinate chloritization of the garnet minerals occurs. Ores are present as accessory minerals.

The slightly formed foliation generally dips very steeply to the north, and the dividing planes formed in the course of emplacement and tectonic overprinting also stand very steeply. From a mineralogical point of view, the rocks in the area consist of quartz, feldspars (plagioclase and potassium feldspar), and mica, occasionally also accompanied by accessory minerals such as kyanite [33]. The rock is exposed to weathering in the near-surface area especially along interfaces and fractures, which in turn influences the rock properties. This leads to mineral transformations, which generally reduce the rock strength.

**Figure 1:**
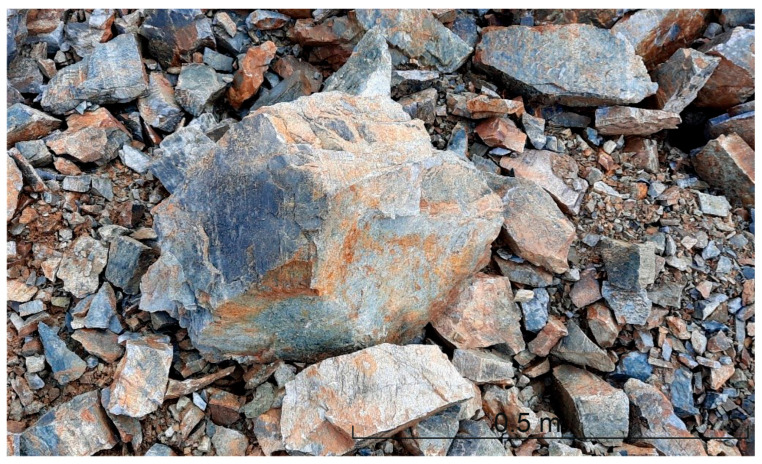
Light-colored, vitreous granulite, corresponding to (1).

**Figure 2:**
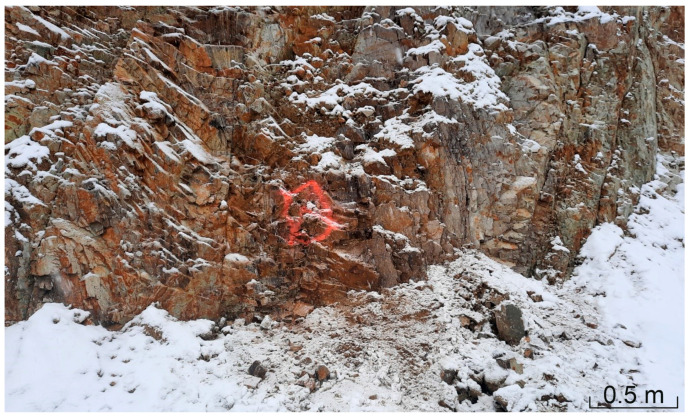
Weathered, strongly jointed granulite, corresponding to (2).

**Figure 3:**
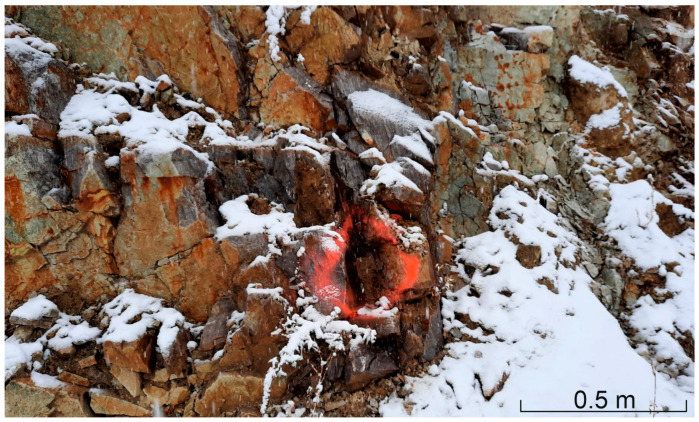
Darker, more deeply lying granulite, corresponding to (3).

Lithologically, the area shows a heterogeneous structure, both in terms of horizontal extent and depth (excavation levels), and is roughly composed of the following different granulite types: (1) the very light-colored unit with a bright sound when crushed (“vitreous granulite”), (2) a brown granulite unit showing stronger weathering signs (highest, weathered area), and (3) a dark, more deeply lying granulite unit. Additionally, serpentinite lenses are situated north and south of the investigated granulite area. Photos of areas (1), (2), and (3) are shown in Figure 1, Figure 2 and Figure 3.

The highest quality granulite rocks (light-colored, “vitreous granulite”) are extracted for the production of aggregates for railroad gravel, concrete, and asphalt. These are characterized by very high compressive strength (≥200 MPa), high impact abrasion and frost resistance. The other granulite rocks are mainly used for the production of base course and fill material. For the quality classification, the regular assessment of the rock properties—primarily by means of the Los-Angeles test—plays an essential role in ensuring the high-quality requirements of the aggregate.

**Figure 4:**
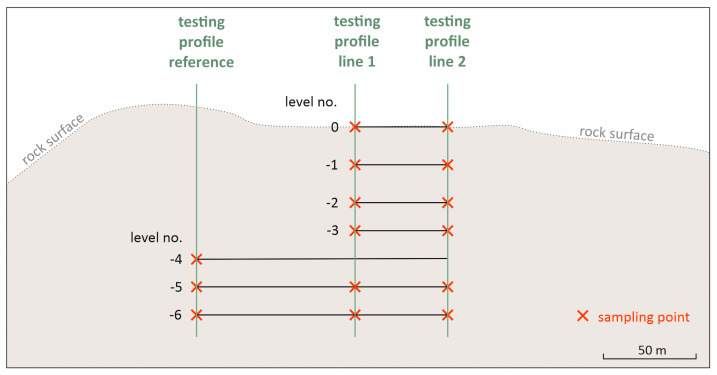
Schematic illustration of the sampling along two vertical measurement lines and of the reference rock along a third vertical measurement line.

**Figure 5:**
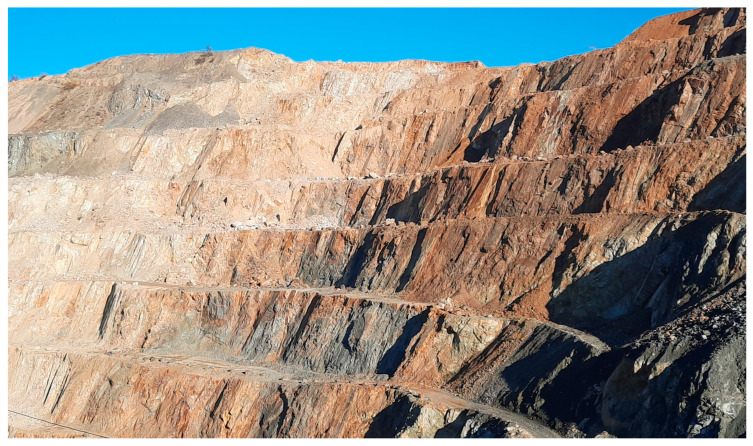
Section of the investigated quarry wall (oblique view); compare Figure 4.

In order to systematically demonstrate the variability of the rock properties, sampling and examination of the granulite bedrock at different depth levels (a total of seven depth levels with generally 15 to 20 m height difference were investigated) took place along two vertical profiles (Figure 4). As a reference, three depth levels in the area of the glassy granulite were also sampled and surveyed (reference testing profile, Figure 4). A partial section of the sampling area is shown in Figure 5. The light coloured, glassy vitreous granulite can be seen in the background, the darker granulite in the front, with the alteration (and thus the brownish discoloration) increasing towards the top of the terrain. The vitreous granulite was used as a reference profile due to its very high and desired strength properties and the values determined were then standardized accordingly.

### 2.2. Testing Methods

In order to record and compare the mechanical rock properties at different sampling positions, the following methods were used:

- the rebound hammer (RH)—or also referred as Schmidt hammer—was used as an indirect non-destructive method for determining the rock strength (derivation of the unconfined compressive strength) [15,16,34];
- the point load test (PLT) was applied as a destructive method to determine the rock strength (convertible to unconfined rock compressive strength) using rock handpieces [17,18,35];
- the Los Angeles test (LAT) as a standard industrial test procedure for the recording of the impact-abrasion-resistance of rock aggregate [5,20,36].

These methods offer the possibility of estimating the uniaxial compressive strength (UCS) as a substitute for the uncon-fined compression test on drill cores. Especially in highly fractured rock, it is not possible to obtain a suitable drill core for a compression test on the rock cylinder. The methods mentioned above offer an indirect possibility of rock strength estimation. On the basis of empirical comparative studies, it is possible to deduce the UCS from the parameters (re-bound value, point load resistance, LA class) obtained by these methods [17,37,38,39].

The methods are presented in detail in the following subchapters. Additionally, X-ray diffraction (using a PANanalytical X’pert PRO from Malvern Panalytical, Malvern, United Kingdom) and thin section analysis (via Leica DM4500P polarization microscope from Leica Microsystems GmbH, Wetzlar, Germany) were applied to exclude mineralogical and structural sources of error.

#### 2.2.1. Schmidt/Rebound Hammer (RH)

The Schmidt rebound hammer is an indirect method for determining the strength of rock or concrete [15,16,40]. Originally designed for the non-destructive testing of concrete hardness, the RH is an index apparatus to measure the surface hardness very quickly and inexpensively. Nowadays the RH represents the most frequently used index method to estimate the uniaxial compressive strength (UCS) of rock and concrete in laboratory as well as in situ [41,42].

**Figure 6:**
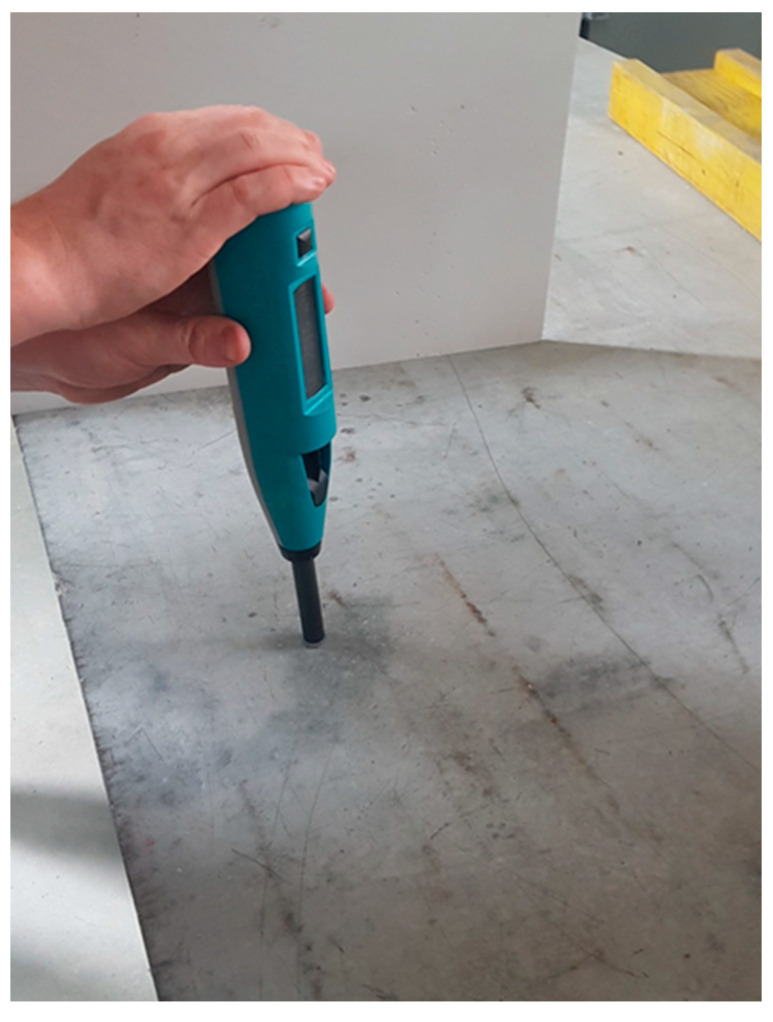
Schmidt hammer when testing the repeatability on a 30 cm thick concrete slab (used as reference material).

Regarding the testing mechanism, the RH is a spring-loaded piston, which is manually prestressed and automatically releases when the tip of the piston is pressed against the surface to be measured (Figure 6). The energy of the spring is released and is then mainly consumed by the plastic deformation of the material under the tip. A part of the energy is rebounded depending on the impact penetration resistance. The harder or stronger the test subsurface, the smaller is the distance travelled by the piston tip and the lower the spring extension. The result of the measurement is a rebound value, measured as a percentage of the initial extension of the spring to the spring extension after testing. This ratio represents an index for surface hardness. This index can be converted into an UCS via empirical relationships for both rock and concrete [15,16]. For this study, however, the index value was used without conversion to a UCS to avoid introducing an uncertainty component via empirical variables.

The Schmidt rebound hardness (“rebound value” *R*) was determined as a non-destructive field test method via the rebound hammer “Rock Schmidt” model N of the company “proceq” (impact energy 2.2 Nm). Testing was carried out according to the specifications of [34]. A total of 20 impact tests were carried out in a narrow rock area at the different sampling points (Figure 4), all measured values of a standard measurement run were included in the statistical evaluation. The same was done comparatively on a high strength concrete slab to check the repeatability (Figure 6).

#### 2.2.2. Point Load Test (PLT)

During the PLT, a rock sample is clamped between two standardized test tips with 60° test point angle and loaded until failure (point load test device type “Wille Geotechnik”), i.a. [17,18,38,39]. The contact area between the cone tips and the specimen are called load application points. The breaking load obtained is called the point load index *Is* [6]. This corresponds to the strength of a rock specimen that breaks under a concentrated applied load. Similar to the Schmidt hammer, there are also empirically determined correlations between point load index and UCS depending on the rock type [43,44,45]. For this study, the initial values (point load index *Is*) were used for the correlation calculations.

Fist-sized rock specimens were used to perform the PLT [17,34]. The point load index Is was calculated according to Equation (6):

(6) $$I_{s}=\frac{P}{D_{e}^{2}}$$

whereby:

*Is*—point load index [MPa],
*P*—load at failure [N],
*D_e_*—equivalent core diameter [mm].

A correction of the sample size to calculate the corrected point load Index *Is*_(50)_ [MPa] was carried out as follows (Equation (7)):

(7) $$I_{s(50)}={\left(\frac{D_{e}}{50}\right)}^{0.45}\cdot I_{s}$$

**Figure 7:**
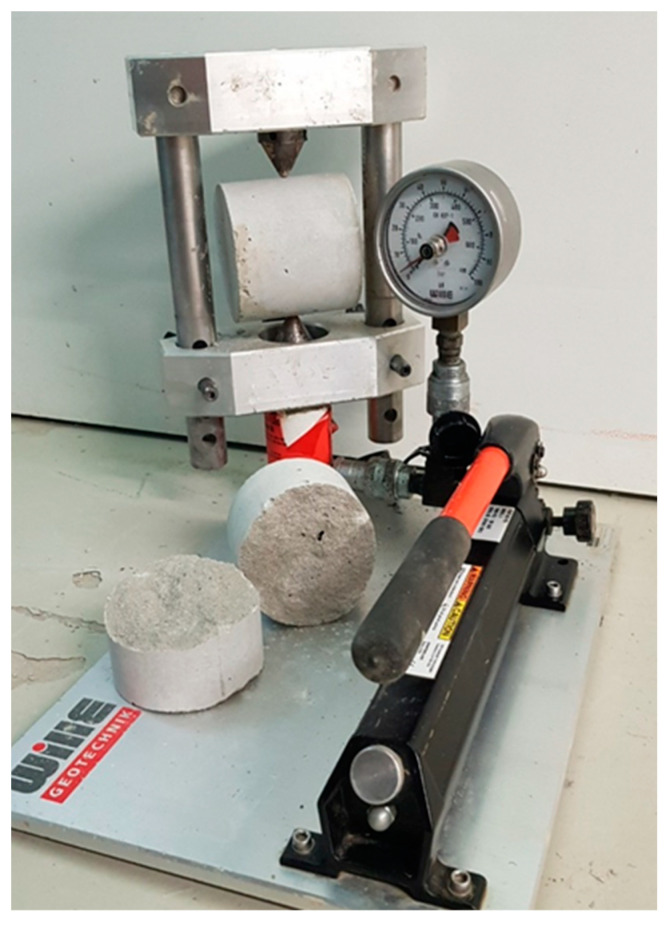
Point load testing on concrete mortar cylinders (used as reference material).

At each sampling point (Figure 4), 30 individual measurements were carried out to account for the high scatter of this measurement method. Comparative tests on concrete mortar specimens were carried out for comparative purposes (Figure 7).

#### 2.2.3. Los Angeles Test (LAT)

**Figure 8:**
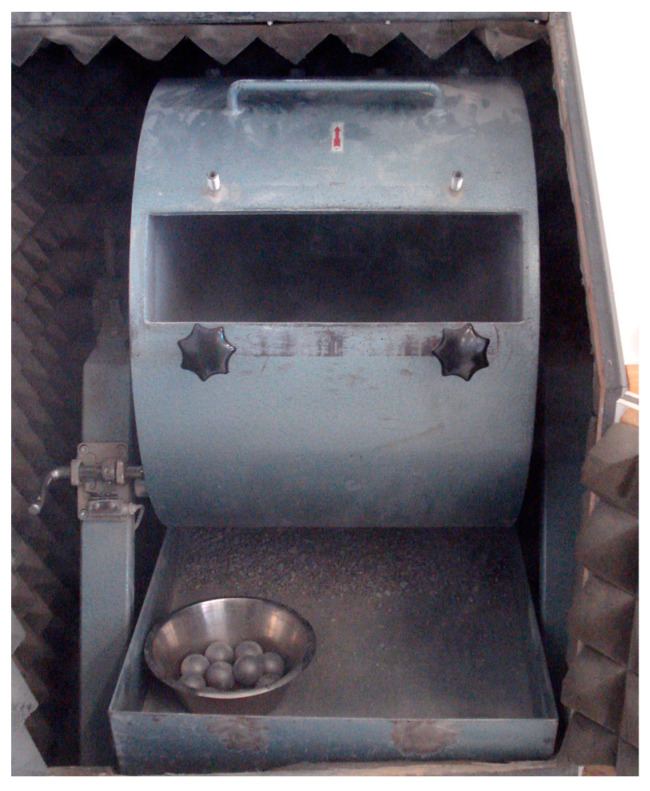
Los Angeles testing machine with steel balls in the front (in soundproof enclosure).

To evaluate the impact abrasion resistance, the LAT was performed according to the standard EN 1097-2 [20] using a Los-Angeles testing device from the manufacturer ’testing’ (Figure 8). At the beginning of the test, a 5 kg (±5 g) rock sample with a grain size of 10 to 14 mm is placed in a rotatable steel drum together with eleven standardized steel balls. The subsequent rotation (32 ± 1 min^−1^) of the drum crushes or abrades the rock sample during 500 rotations. The LA value corresponds to the passing of the tested material through a 1.6 mm sieve [5,20]. Accordingly, the higher the percentage of sieve passage smaller than 1.6 mm, the less abrasion resistant the rock sample is as an expression of mechanical resistance to impact and abrasion exposure [20]. The UCS of a rock can also be derived from the LA value by means of empirically determined correlations [46].

### 2.3. Contributions on the Budget of Measurement Uncertainty

**Figure 9:**
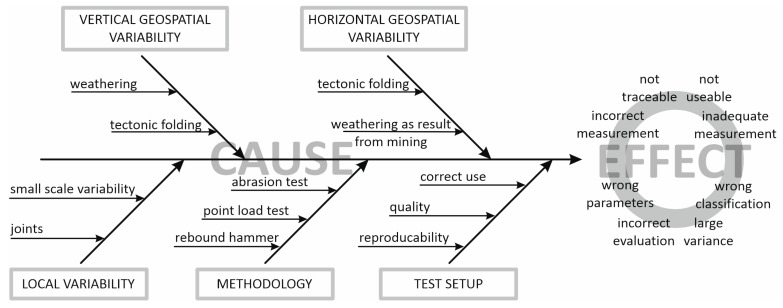
Cause-and-effect diagram for the determination of the influences on the measurement uncertainty of rock material parameters.

In accordance with the general explanations provided in Section 1.3, a model for the influences on the uncertainty for the determination of rock material parameters can be established. As target value the material parameter of rebound value (*R*) as well as the point load index (*Is*_(50)_) and the Los-Angeles-value (*LA*-value) from the Los Angeles fragmentation test are considered. The determination of this model and the influences on the model are based on a discussion process. Figure 9 shows the investigated influences resp. error causes on the measurement on the left side by means of a cause-and-effect diagram, as well as the obtained effects on the measurement in accordance to [47].

The following influences on the uncertainty of the measurement have been identified in Figure 9 and are discussed below.

- Geospatial variability: large-scale variability considering the spatial variability of the rock as a result of:
  (a) the weathering processes progressing vertically downwards from the top level which is exposed to the environment;
  (b) the weathering processes caused by exposed surfaces as a result of mining;
  (c) the geological variability, which can vary vertically and horizontally in consequence of the rock formation processes.
- Small scale variability: variation in the rock in the small range as a result of the different (micro)structural conditions: dividing planes (e.g., joints) and the resulting inherent uncertainties.
- Methodology: the applied methods such as rebound hammer method, point load test, and fragmentation test, as well as the uncertainties coming from the used method itself including the repeatability.
- Test setup: the quality and traceability of the used measurement devices, correct technical use and in the appropriate measurement range influencing the reproducibility.

Hence, as a result of the findings above, the model of the influences on the measurement uncertainty might be defined in analytical form as following (Equation (8)).

(8) $$Y=f\left(u_{var,hor},u_{var,vert},u_{method},u_{repeatability},u_{reproducability}\right)$$

in which:

*u_var,hor_*—uncertainty due to geological variability in horizontal direction of the rock mass (as a result of the site only relevant in this direction),
*u_var,ver_*—uncertainty due to weathering processes progressing vertically downwards from the top level resulting from environmental influences,
*u_method_*—uncertainty due to different testing methodologies,
*u_repeatability_*—uncertainty considering the repeatability of test results under ideal conditions,
*u_reproducability_*—uncertainty considering the reproducibility of test results under real conditions.

The model from Equation (8) is considered as a basic theoretical consideration. Subsequently, individual components are quantified on the basis of available data and evaluated as to whether they should be considered or not.

## 3. Results and Evaluation

### 3.1. General

This section presents the results of the conducted investigations as described above and their evaluation. The geological formation presented in Section 2.1 was analyzed regarding its mechanical rock parameters using the testing methods according to Section 2.2. Section 3.2 provides the test results, and Section 3.3 a detailed approach for the estimation of the measurement uncertainty of the determination of the rock properties.

### 3.2. Test Results

The investigation area consists of a total of seven exposed levels over a total depth of approximately 100 m, which means 15 m to 20 m between each horizon (Figure 4). An overview of the performed tests is shown in Table 1.

**Figure 10:**
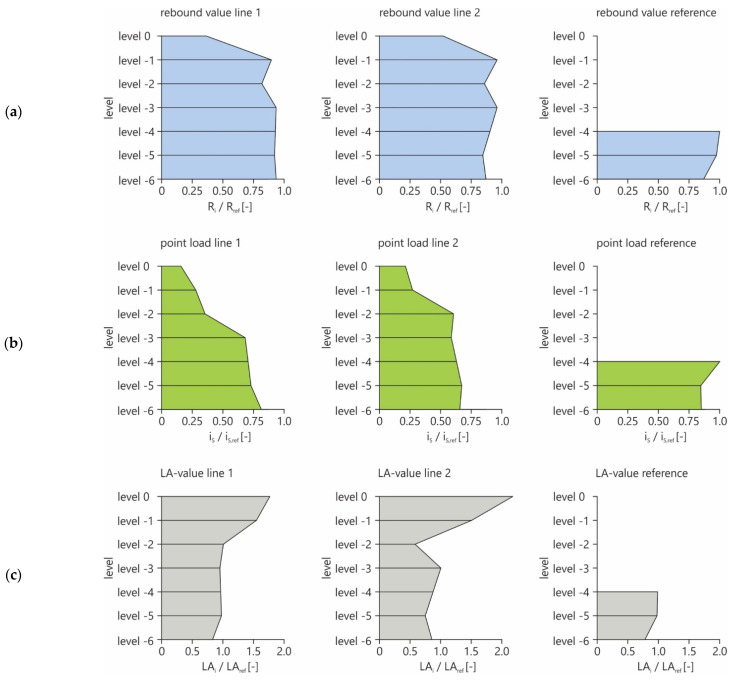
Overview of the test results of (**a**) rebound values, (**b**) point load tests and (**c**) Los Angeles test referred to the maximum value of level −4 for each considered material parameter.

The results of the performed tests as the rebound value (*R*), the *Is*_(50)_ from the PLT and the *LA* value are shown in Figure 5. The tests were performed in two vertically aligned measurement lines (line 1 and line 2). Additionally, the reference values were determined in more or less undisturbed rock at measuring points in the measuring horizons −4 to −6, which were considered to be unweathered a priori due to their geological conditions (Figure 1). Level −4 showed the highest rock quality with the highest material properties and therefore was set as the reference value to which all measured values of the particular testing methods in the presentation are referred. Accordingly, the values shown in Figure 10 are to be regarded as percentages of the respective reference value, whereby the *LA* value behaves reciprocally. Figure 10 shows the normalized ratio of the respective test results (test respective test results were normalized to the values of the reference rock) for the different test profiles (cf. Figure 1) and the reference rock material for the respective levels. As reference value, the maximum value of the reference measurement (level −4) has been considered. In Figure 10, a structured overview of the distribution of the various parameters of the rock over the depth of the measured lines is presented.

As shown in Figure 10, there are considerable differences in the test results, which are evident as follows:

- the top horizon or the two top horizons show the lowest values for both the RH and the PLT, i.e., material damage is evident here. In the case of the LAT, the highest values are reached in these levels, which also indicates increasing material damage, which causes increased fragmentation. This is clearly due to the influences of the environmental conditions (weathering processes).
- The two vertical measurement lines do not indicate any clearly identifiable differences in the corresponding levels, so it can be assumed that the two measurement lines are comparable.
- The reference measurements in levels −4 to −6 (high value grain of the quarry) show values that indicate a high-quality rock material as these are within the range of the reference value.

### 3.3. Estimation of Measurement Uncertainty for the Determination of Rock Material Parameters

The general principles for determining the contributions to the measurement uncertainty budget are stated in Section 1.3. Following the determination of the measurement uncertainty, this is derived for the three test methods that were performed.

#### 3.3.1. Measurement Uncertainty for Determination of Rebound Value

For the determination of the rebound value *R*, the contributions for the measurement uncertainty have been considered as listed in Equation (8). The estimation of the individual quantities is conducted as follows (Table 2):

The expanded uncertainty of measurement for the rebound value has a value of approximately 20% to 30% depending on whether the weathering effects in the two upper horizons are considered or not.

#### 3.3.2. Measurement Uncertainty for Determination of Uniaxial Compressive Strength from Point Load Test

For the determination of the corrected point load index *Is*_(50)_ from point load test, the contributions for the measurement uncertainty have been considered as listed in Equation (8) with estimation of the quantities as follows (Table 3):

The value for the expanded uncertainty of measurement for the corrected point load index *Is*_(50)_ ranges from approximately 40% to 116% depending on whether the weathering effects in the two upper horizons are considered or not.

#### 3.3.3. Measurement Uncertainty for Determination of LA Value from Fragmentation Test

For the determination of the *LA* value from fragmentation testing, the contributions for the measurement uncertainty are as follows (Table 4):

The expanded uncertainty of measurement for the *LA* value from fragmentation testing lies between approximately 60% and 120% depending on whether the weathering effects in the two upper horizons are considered or not.

#### 3.3.4. Overview of Measurement Uncertainty

**Figure 11:**
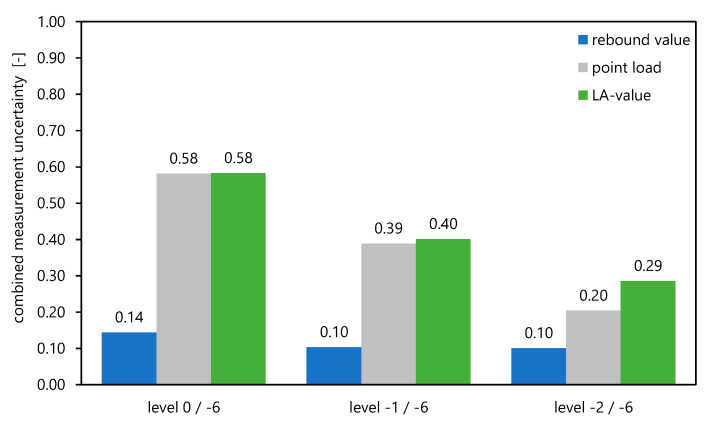
Comparison of combined measurement uncertainty for different testing methods.

Based on the evaluation of the test results in the sections above, the combined measurement uncertainty *u* for the different measurement methods is graphically shown in Figure 11 based on the values from Table 2, Table 3 and Table 4. The uncertainties differ significantly depending on the investigated rock level. There is a considerable difference within the combined measurement uncertainty between the in-situ methods, which are the rebound value and the point load test, resulting also because of the different number of conducted repeated test series. In the case of a larger number of measurements there is a higher certainty that the received value is true. The *LA* value is to be considered as the decisive parameter for the assessment of the suitability of the material. It is evident that the point load index *Is*_(50)_ in Figure 11 reflects similar combined measurement uncertainties as the *LA* values as a result of the comparable number of test series.

**Figure 12:**
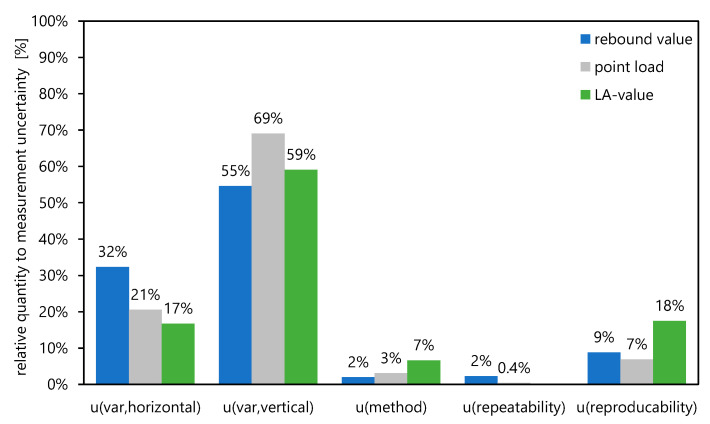
Percentage contribution of the different input variables on the total measurement uncertainty for different testing methods.

The single contributions to the measurement uncertainty shown in Table 2, Table 3 and Table 4 are finally referenced to the determined combined uncertainty (all contributions subsequently represent 100%) deriving the percentage contribution of the different input variables as shown in Figure 12.

It is obvious that the percentages of the vertical variability represent the majority of the total measurement uncertainty. Therefore, it is obvious that is necessary and also technically sensible to distinguish areas with evidence of weathering from those with no evidence of weathering. This reduces the uncertainty of the measurement by up to 70%.

As already stated in Table 2, Table 3 and Table 4, the measurement uncertainty can be significantly reduced if weathered and unweathered rocks are considered separately.

For example, a reduction in the combined measurement uncertainty from 0.14 to 0.10 can be observed for the rebound value *R*, from 0.58 to 0.20 for the *Is*_(50)_ from the point load test and from 0.58 to 0.29 for the *LA* value if the two upper weathered horizons are considered separately as shown in Table 5.

Additionally, as indicated in Table 5, if the horizontal geospatial variation can be a priori considered, the combined measurement uncertainty is reduced for the different testing methods to the following values: 0.13 to 0.07 for the re-bound value *R*, 0.57 to 0.13 for the *Is*_(50)_ from the point load test, and 0.57 to 0.24 for the *LA* value.

## 4. Conclusions

In general, uncertainties in the measurement method are difficult to determine quantitatively, if no repeated measurements are feasible. Specifically, this is the case in the determination of parameters of natural materials that are subject to non-reproducible influences. For the validity of a measurement, the knowledge of the measurement uncertainty is essential. This is particularly the case when compliance with limit values or technical specifications is considered.

The measurement uncertainty itself therefore has a considerable influence on the significance and the reliability of measurements. This also applies to the methods for the determination of the rock characteristics in geology. In this publication, analyses are presented on the estimation of the measurement uncertainty in the determination of material parameters of natural rock mass.

The following conclusions can be made:

- the combined measurement uncertainty of the compared testing methods shows that, as is generally known, the measurement uncertainty decreases with an increasing number of tests, and the real value can be approximated with a higher accuracy;
- it is obvious that a consideration of a priori information as the different horizons/levels is a method to reduce the combined uncertainty *u*; this can be reduced to values of approximately 10–30% depending on the testing method and the number of test series also considering the weathering effects;
- as shown in Figure 10, the rebound value *R* is not considering the effects from weathering in the same way as the point load index *Is*_(50)_ and the *LA* value; this approach therefore only has restricted significance.
- the relative influence on the combined uncertainty *u* of the different testing methods shows a range of *u_var,hor_* between 17% and 32% due to the geological variability in the horizontal direction; in comparison, the re-bound hammer method has the highest relative measurement uncertainty;
- the relative influence on the combined uncertainty reaches values for *u_var,ver_* from 55% to 69% as a result of the different vertical direction (levels) due to weathering phenomena at the surface;
- considering the vertical direction, the point load test shows the highest contribution to the relative quantity of measurement uncertainty with a value of 69%; however, such influence is quite comparable within all measurement methods;
- this leads to the conclusion that a higher weathering degree of the rock has an increasing effect on the measurement uncertainty; therefore, the use of a priori information for the realization of a testing task in rock is strongly recommended;
- weathering can affect both the discontinuity itself and the surrounding rock.

In terms of aggregate production, weathering of the discontinuity surface is almost unproblematic, but the progress of weathering into the surrounding rock results in a reduction in the rock (aggregate) strength. Starting from the surface level, weathering must always be considered as an influencing factor; this was also clearly observed in the present study. The observed variability generally implies a significant challenge in the assessment of the associated material parameters.

These influences are inadequately reflected by the RH. While the ‘aleatoric’ uncertainty is predetermined by the rock mass structure and cannot be statistically considered, the test method-related uncertainty increases the ‘epistemic’ part of the total uncertainty and thus the overall uncertainty. This means that if only the RH is used to assess the mechanical rock parameters during an on-site rock mass testing (this concerns both the addressing of rock in an existing quarry, as well as in investigations for the expansion of the quarry), it is possible that the quality of the aggregates produced from the rock mass will not be sufficient. Accordingly, it is strongly recommended by the authors to verify the rebound values by test results of the in-situ feasible PLT or the LAT in the laboratory. These more complex, but more meaningful test methods are associated with a significantly lower measurement uncertainty and are therefore recommended for the considered applications.

## Figures and Tables

**Table 1:** Overview of the considered tests for rock material parameters.

Testing Method	Line	Testing Horizons Resp. Levels	n = Number of Tests at Each Location
Rebound hammer	1	0/−1/−2/−3/−4/−5/−6	3 × 10
	2	0/−1/−2/−3/−4/−5/−6	3 × 10
	reference	−4/−5/−6	3 × 10
Point load test	1	0/−1/−2/−3/−4/−5/−6	1 × 30
	2	0/−1/−2/−3/−4/−5/−6	1 × 30 ^1^
	reference	−4/−5/−6	1 × 30
LA-test	1	0/−1/−2/−3/−4/−5/−6	1 × 1
	2	0/−1/−2/−3/−4/−5/−6	1 × 1
	reference	−4/−5/−6	1 × 1

^1^ in horizon −6 for comparison reasons two independent test series were performed.

**Table 2:** Estimation of measurement uncertainty for determination of rebound value.

Uncertainty	Description	Calculation	Valid for	n	*u_mean_*	*u_median_*	Min.Value *u*	Max.Value *u*
			[-]	[-]	[-]	[-]	[-]	[-]
*u_var,hor_*	uncertainty due to geological variability of the rock mass in horizontal direction	$\left(\frac{\left|R_{ref,m}-R_{i,m}\right|}{R_{ref,m}}\right)$	all levels	3	0.07	0.07	0.03	0.10
*u_var,ver_*	uncertainty due to weathering processes progressing vertically downwards from the top level resulting from environmental conditions	$\left(\frac{\left|R_{level\;i}-R_{level\;j}\right|}{R_{level\;i}}\right)$	all levels	30 × 10	0.22	0.12	0.001	0.61
			level −1/−6	20 × 10	0.07	0.07	0.001	0.14
			level −2/−6	10 × 10	0.07	0.07	0.001	0.14
*u_method_*	uncertainty due to different testing methodologies tested on reference material	$\sqrt{\frac{1}{n\left(n-1\right)}\sum_{i=1}^{n}{\left(R_{i}-R_{m}\right)}^{2}}$	all levels	40	0.004	0.004	no value	no value
*u_repeatability_*	uncertainty considering the repeatability of test results under ideal conditions	$\sqrt{\frac{1}{n\left(n-1\right)}\sum_{i=1}^{n}{\left(R_{i}-R_{m}\right)}^{2}}$	all levels	30 × 10	0.006	0.005	0.001	0.02
*u_reproducability_*	uncertainty considering the reproducability of test results under real conditions	$\frac{CV}{\sqrt{n}}$	all levels	30 × 10	0.05	0.05	0.01	0.18
$u_{c}$	combined standard uncertainty in accordance with Equation (3)		all levels	0.14				
			level −1/−6	0.10				
			level −2/−6	0.10				
$U=k\cdot u_{c}$	expanded measurement uncertainty using *k* = 2		all levels	0.29				
			level −1/−6	0.21				
			level −2/−6	0.20				

**Table 3:** Estimation of measurement uncertainty for determination of uniaxial compressive strength from point load test.

Uncertainty	Description	Calculation	Valid for	n	*u_mean_*	*u_median_*	Min.Value *u*	Max.Value *u*
			[-]	[-]	[-]	[-]	[-]	[-]
*u_var,hor_*	uncertainty due to geological variability of the rock mass in horizontal direction	$\left(\frac{\left|{I_{s(50)}}_{ref,m}-{I_{s(50)}}_{i,m}\right|}{{I_{s(50)}}_{ref,m}}\right)$	all levels	3	0.19	0.17	0.13	0.28
*u_var,ver_*	uncertainty due to weathering processes progressing vertically downwards from the top level resulting from environmental influences	$\left(\frac{\left|{I_{s(50)}}_{level\;i}-{I_{s(50)}}_{level\;j}\right|}{{I_{s(50)}}_{level\;i}}\right)$	all levels	30 × 10	0.42	0.52	0.02	0.79
			level −1/−6	20 × 10	0.33	0.34	0.02	0.63
			level −2/−6	10 × 10	0.19	0.12	0.02	0.52
*u_method_*	uncertainty due to different testing methodologies tested on reference material	$\sqrt{\frac{1}{n\left(n-1\right)}\sum_{i=1}^{n}{\left({I_{s(50)}}_{i}-{I_{s(50)}}_{m}\right)}^{2}}$	all levels	20	0.025	0.025	no value	no value
*u_repeatability_*	uncertainty considering the repeatability of test results under ideal conditions	$\sqrt{\frac{1}{n\left(n-1\right)}\sum_{i=1}^{n}{\left({I_{s(50)}}_{i}-{I_{s(50)}}_{m}\right)}^{2}}$	all levels	2 × 10	0.003	0.003	no value	no value
*u_reproducability_*	uncertainty considering the reproducability of test results under real conditions	$\frac{CV}{\sqrt{n}}$	all levels	15 × 10	0.05	0.05	0.03	0.08
$u_{c}$	combined standard uncertainty in accordance with Equation (3)		all levels	0.58				
			level −1/−6	0.39				
			level −2/−6	0.20				
$U=k\cdot u_{c}$	expanded measurement uncertainty using *k* = 2		all levels	1.16				
			level −1/−6	0.78				
			level −2/−6	0.41				

**Table 4:** Estimation of measurement uncertainty for determination of *LA*-value from fragmentation testing.

Uncertainty	Description	Calculation	Valid for	n	*u_mean_*	*u_median_*	Min.Value *u*	Max.Value *u*
			[-]	[-]	[-]	[-]	[-]	[-]
*u_var,hor_*	uncertainty due to geological variability of the rock mass in horizontal direction	$\left(\frac{\left|{LA}_{ref,m}-{LA}_{i,m}\right|}{{LA}_{ref,m}}\right)$	all levels	3	0.11	0.15	0.02	0.17
*u_var,ver_*	uncertainty due to weathering processes progressing vertically downwards from the top level resulting from environmental influences	$\left(\frac{\left|{LA}_{level\;i}-{LA}_{level\;j}\right|}{{LA}_{level\;i}}\right)$	all levels	30 × 10	0.67	0.54	0.02	2.74
			level −1/−6	20 × 10	0.43	0.33	0.02	1.58
			level −2/−6	10 × 10	0.19	0.17	0.02	0.42
*u_method_*	uncertainty due to different testing methodologies tested on reference material	$\sqrt{\frac{1}{n\left(n-1\right)}\sum_{i=1}^{n}{\left({LA}_{i}-{LA}_{m}\right)}^{2}}$	all levels	no value ^1^	0.06	0.06	no value	no value
*u_repeatability_*	uncertainty considering the repeatability of test results under ideal conditions	$\sqrt{\frac{1}{n\left(n-1\right)}\sum_{i=1}^{n}{\left({LA}_{i}-{LA}_{m}\right)}^{2}}$	no value	no value ^1^	no value	no value	no value	no value
*u_reproducability_*	uncertainty considering the reproducability of test results under real conditions	$\frac{CV}{\sqrt{n}}$	all levels	no value	0.16	0.16	no value	no value
$u_{c}$	combined standard uncertainty in accordance with Equation (3)		all levels	0.58				
			level −1/−6	0.40				
			level −2/−6	0.29				
$U=k\cdot u_{c}$	expanded measurement uncertainty using *k* = 2		all levels	1.17				
			level −1/−6	0.80				
			level −2/−6	0.57				

^1^ in acc. to EN 1097-2.

**Table 5:** Overview of the calculated combined uncertainty of measurement *u* for different conditions.

Expanded Measurement Uncertainty U for Testing Method	A Priori Consideration of Vertical Variation		A Priori Consideration of Horizontal Variation	
	All Levels Including Weathering	Level −2/−6 No Weathering	All Levels Including Weathering	Level −2/−6 No Weathering
RH	0.14	0.10	0.13	0.07
PLT	0.58	0.20	0.57	0.13
LAT	0.58	0.29	0.57	0.24

## Data Availability

The raw/processed data required to reproduce these findings cannot be shared at this time due to legal or ethical reasons.
